# Extensive spontaneous intramural hematoma of rectum and sigmoid colon in a patient undergoing anticoagulant therapy: A case report

**DOI:** 10.1097/MD.0000000000042428

**Published:** 2025-05-30

**Authors:** Joanna Kula, Paweł Szmigiel, Cezary Rusinowski, Sławomir Mrowiec, Piotr Wosiewicz

**Affiliations:** a Department of Internal, Autoimmune and Metabolic Diseases, Faculty of Medical Sciences in Katowice, Medical University of Silesia, Katowice, Poland; b Department of Digestive Tract Surgery, Faculty of Medical Sciences in Katowice, Medical University of Silesia, Katowice, Poland; c Department of Gastroenterology and Hepatology, Faculty of Medical Sciences in Katowice, Medical University of Silesia, Katowice, Poland.

**Keywords:** gastrointestinal bleeding, intramural hematoma, rectum, sigmoid colon

## Abstract

**Rationale:**

Intramural hematoma of the large intestine is a rare and potentially life-threatening condition. Nonspecific clinical symptoms pose a diagnostic challenge, necessitating prompt recognition for timely and appropriate management. The selection of an optimal therapeutic approach depends on multiple factors, including the etiology and severity of bleeding, the patient’s overall condition, and the availability of various treatment modalities.

**Patient concerns:**

This case report describes a 67-year-old male admitted for acute abdominal pain and hematochezia persisting for several hours.

**Diagnoses:**

Sigmoidoscopy revealed a large, smooth-walled, bluish-purple mass nearly occluding the rectum, suggestive of an intramural hematoma. Multidetector contrast-enhanced computed tomography of the abdomen and pelvis confirmed an extensive intramural hematoma of the rectum and sigmoid colon with active bleeding.

**Interventions:**

The patient underwent emergency surgery, during which the massive hematoma was evacuated. Hemostasis was achieved using seton placement and a diverting loop ileostomy. On postoperative day 2, the setons were removed, and reassessment confirmed the absence of active bleeding. The patient’s condition gradually improved. Hematological parameters remained stable throughout the follow-up.

**Outcomes:**

On the 10th postoperative day, the patient was discharged in good general condition with a well-healing surgical wound. Ileostomy reversal was performed 6 months after the initial hemorrhagic event, and the patient was subsequently discharged in good general condition without any complications and clinical consequences. An unconventional surgical strategy involving hematoma evacuation without intestinal resection preserved physiological bowel function, thereby preventing adverse clinical consequences.

**Lessons:**

This report highlights a case of extensive, spontaneous intramural hematoma of the rectum and sigmoid colon in a patient receiving anticoagulant therapy. Although rare, these hematomas should be considered in the differential diagnosis of abdominal pain in anticoagulated patients. A timely diagnosis based on clinical evaluation, thorough physical examination, and imaging studies is crucial for appropriate management. In this case, early diagnosis facilitated surgical intervention while preserving intestinal continuity.

## 1. Introduction

Intramural hematomas are a rare clinical condition. They are commonly linked to blunt abdominal trauma but can also develop spontaneously in patients receiving anticoagulant therapy or those with a bleeding tendency. Clinical manifestations of colonic intramural hematomas may include abdominal pain, lower gastrointestinal bleeding, and, in some cases, bowel obstruction. Computed tomography (CT) and colonoscopy allows rapid and accurate diagnosis. Standard management includes conservative, endoscopic, endovascular and surgical treatment, as well as their combination. We present a case of extensive spontaneous intramural hematoma of rectum and sigmoid colon in a 67-year-old male undergoing anticoagulant therapy along with a literature review.

## 2. Case report

A 67-year-old patient in moderate general condition, pale, with increasing weakness but maintaining logical contact, was presented to the hospital with hematochezia persisting for the last 24 hours accompanied by acute-onset and progressively worsening lower abdominal pain. The patient denied any history of abdominal trauma. His medical history included arterial hypertension, dyslipidemia, deep vein thrombosis of the left lower limb, and pacemaker implantation for second-degree atrioventricular block. He was receiving chronic pharmacotherapy, including acenocoumarol (administered many years ago) ramipril, amlodipine, bisoprolol, eplerenone, acetylsalicylic acid and atorvastatin.

On admission, the patient was hemodynamically stable (blood pressure 120/70 mm Hg, heart rate 72/min). His initial hemoglobin level was 14.5 g/dL (reference range, 13.5–16.5 g/dL), red blood cell count was 4.4 × 10^6^/μL (reference range, 4.2–5.7 × 10^6^/μL), white blood count was 13.98 × 10^3^/μL (reference range, 4.0–10.0 × 10^3^/μL), platelet count was 193 × 10^3^/μL (reference range, 130–400 × 10^3^/μL), hematocrit was 41.5% (reference range, 40%–53%), activated partial thromboplastin time was 29.8 seconds (reference range, 25.4–36.9 seconds), prothrombin time was 27.2 seconds (reference range, 9.4–12.5 seconds), international normalized ratio was 2.27 (reference range, 0.80–1.20), and prothrombin activity was 31% (reference range, 90%–120%), C-reactive protein was 5.5 mg/L (reference range < 5.0).

Physical examination revealed tenderness in the lower abdomen without peritoneal signs, and peristalsis was present. Digital rectal examination detected a large, smooth, firm mass occupying the rectal ampulla.

**Figure 1.**
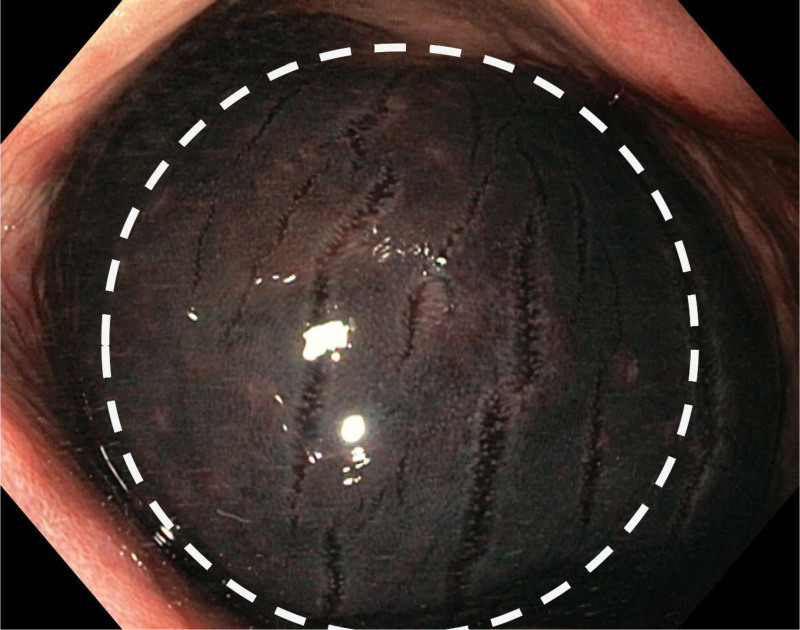
Extensive intramural hematoma filling the lumen of the rectum (white circle).

**Figure 2.**
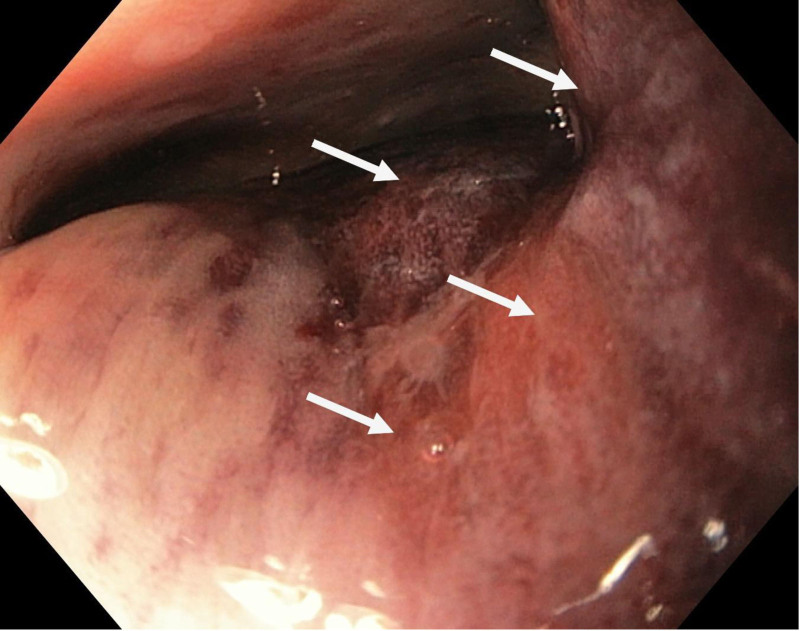
Extensive intramural hematoma filling the lumen of the sigmoid colon (white arrows).

**Figure 3.**
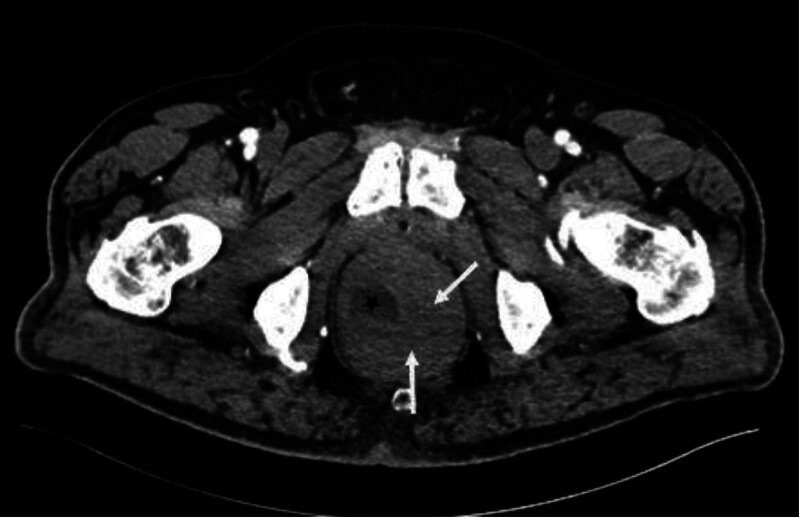
Large rectal hematoma (white arrows) visible on computed tomography image. The lumen of the rectum is marked with a black star.

**Figure 4.**
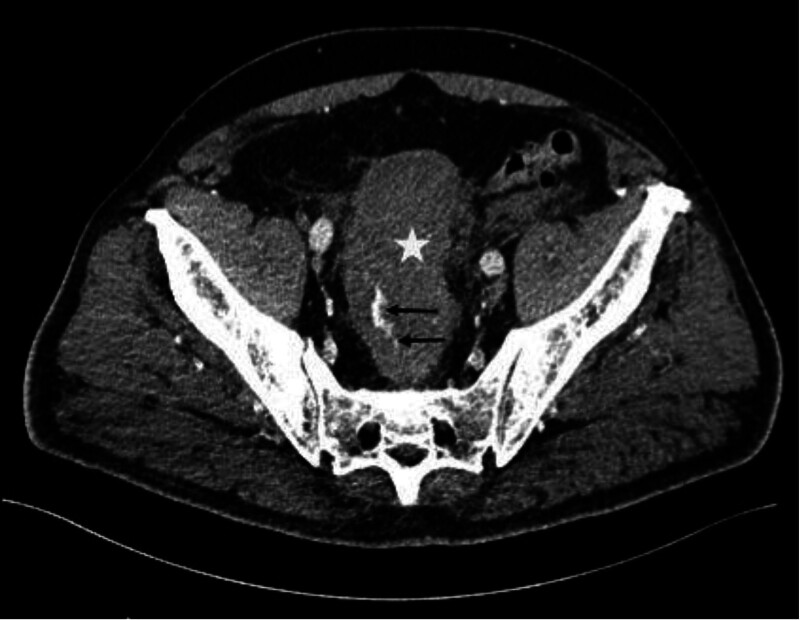
Active bleeding (black arrow) into the lumen of the hematoma (white star) seen on computed tomography scan.

Urgent sigmoidoscopy revealed a large, oval, smooth-walled, bluish-purple structure nearly occluding the rectum, consistent with an intramural hematoma (Fig. 1). Notably, the lesion increased in size during the procedure (Fig. 2). Urgent contrast-enhanced CT confirmed an extensive intramural hematoma in the rectum and sigmoid colon with active bleeding (Figs. 3 and 4). The patient was subsequently qualified for emergency surgical intervention.

During surgery, a massive intramural hematoma of the rectum and sigmoid colon was evacuated. Surgical hemostasis was achieved using seton placement and a diverting loop ileostomy. The procedure was conducted in a manner that preserved the continuity of the sigmoid colon and rectum, thereby avoiding the need for segmental resection.

On postoperative day 2, under operating room conditions, the setons were removed, and reassessment confirmed the absence of active bleeding. The patient’s condition gradually improved, and hematological parameters remained stable during follow-up.

**Figure 5.**
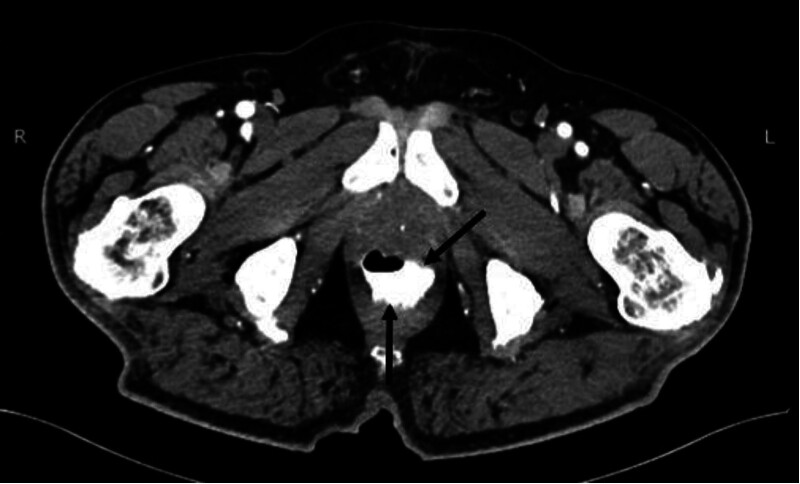
Follow-up CT scan taken 7 weeks postoperatively showing complete resolution of the rectal hematoma, shown in Figure 3. (black arrows). CT = computed tomography.

**Figure 6.**
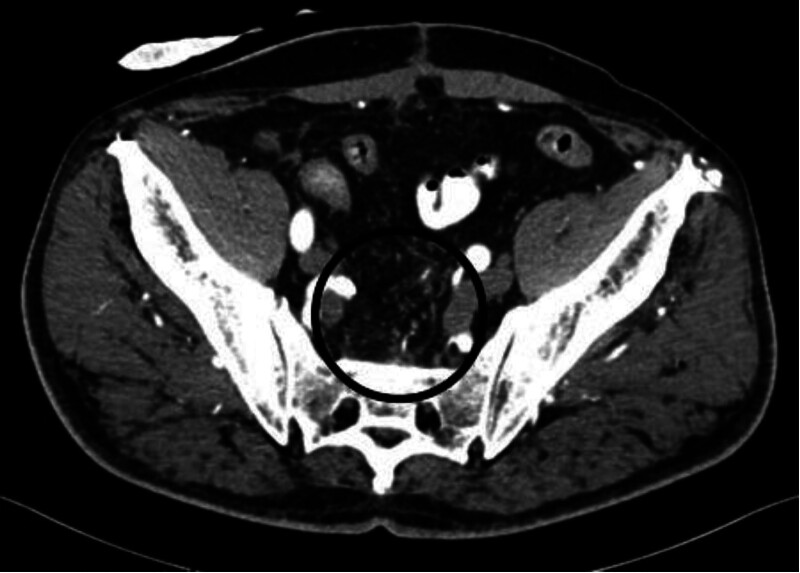
Follow-up CT scan taken 2 months postoperatively showing complete resolution of the hematoma, shown in Figure 5. (black circle). CT = computed tomography.

**Figure 7.**
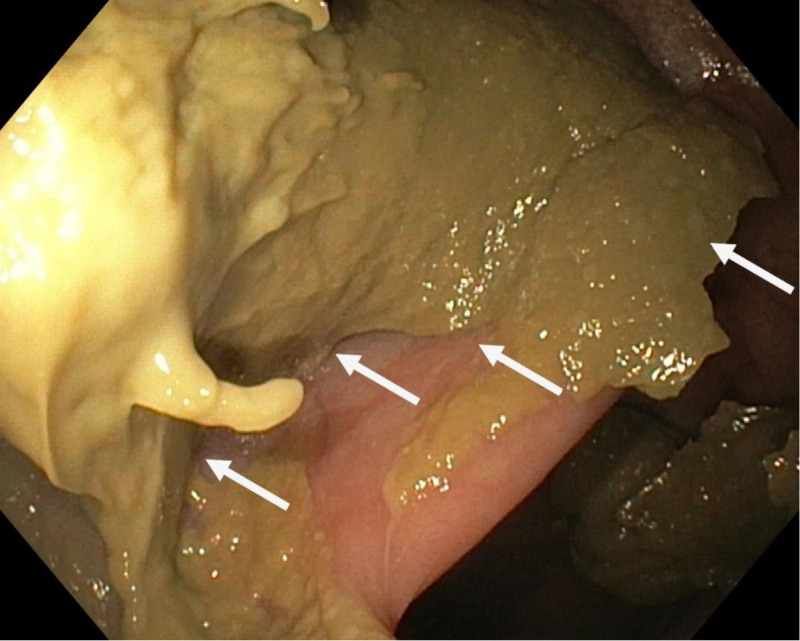
Follow-up colonoscopy 5 months after evacuation of the hematoma. Features of diversion colitis, including retained grayish mucus (white arrows).

**Figure 8.**
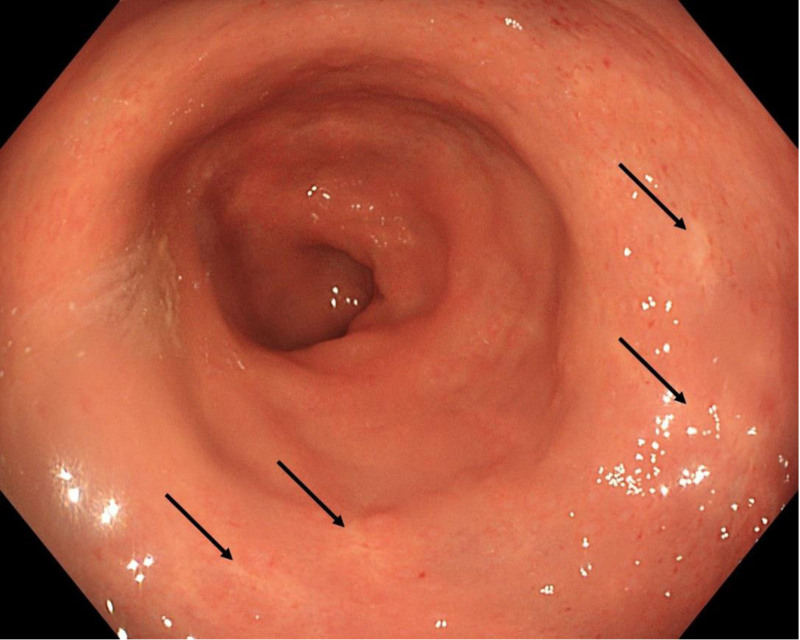
The same patient 5 months after evacuation of the hematoma. Essentially normal rectal mucosa, with minor granulation, petechiae and scarring visible (black arrows).

On the 10th postoperative day, the patient was discharged in good general condition with a well-healing surgical wound. A follow-up contrast-enhanced CT scan performed 2 months later revealed segmental, irregular thickening of the rectal wall without residual hematoma (Figs. 5 and 6). During the observation period, the patient felt well and did not report any complaints. The laboratory tests, including hemoglobin levels, were within normal limits. 5 months postoperatively, a colonoscopy revealed features consistent with diversion colitis, including retained grayish mucus (Fig. 7). Numerous deep diverticula of the sigmoid colon were also noted. At the site of the rectal hematoma only minor scarring was visible with slight granulation and petechiae of the mucosa (Fig. 8). Ileostomy reversal was performed 6 months after the initial hemorrhagic event, and the patient was subsequently discharged in good general condition without complications and clinical consequences.

## 3. Discussion

Intramural hematoma of the colon is a rare entity, typically presenting with key clinical manifestations including gastrointestinal bleeding, progressive secondary anemia, abdominal pain, and, in severe cases, bowel obstruction with hemorrhagic shock. The most common cause is blunt abdominal trauma, particularly in patients receiving anticoagulant therapy or those with coexisting bleeding disorders.^[1]^ Cases have also been reported in patients with colorectal malignancies, in the absence of trauma or anticoagulation therapy.^[2]^ Idiopathic intramural hematomas of the colon are exceedingly rare.^[3–5]^ Among anticoagulants, low-molecular-weight heparin and warfarin are associated with the highest risk of hematoma formation. Achieving a stable therapeutic international normalized ratio is complex and influenced by multiple factors, including warfarin or acenocoumarol dosage and drug interactions. Direct oral anticoagulants selectively inhibit thrombin (dabigatran) or factor Xa (apixaban, edoxaban, rivaroxaban) and are associated with fewer drug interactions than vitamin K antagonists.^[6,7]^

Gastrointestinal hematomas occur in approximately 1 in 250,000 patients receiving anticoagulant therapy.^[8]^

CT with oral and intravenous contrast and colonoscopy are primary diagnostic modalities for intramural hematomas of the colon.^[9]^ Endoscopic ultrasonography provides additional information on wall thickness, extent of involvement, and adjacent tissue damage.^[10]^

Management strategies depend on etiology, hematoma size, clinical symptoms, and patient stability. Conservative management, including anticoagulation reversal, parenteral nutrition, and intravenous hydration, is preferred for hemodynamically stable patients but necessitates temporary suspension of anticoagulation, increasing thromboembolic risk.^[11,12]^

Endoscopic techniques, such as hemostatic clip application, mucosal incision with hematoma evacuation, and ultrasound-guided fine-needle aspiration, offer less invasive alternatives to surgery.^[10]^

Transcatheter arterial embolization is highly effective, with a success rate of up to 85%, but carries a 20% complication rate, including perforation, ischemia, and intestinal wall necrosis.^[10,13]^

Surgical resection is the standard approach for hemodynamically unstable patients or those with complications such as peritonitis, intestinal wall necrosis, obstruction, or perforation.^[14]^ A decompressive colostomy is required in up to 80% of cases.^[10,15]^ Early diagnosis and appropriate conservative or minimally invasive interventions may prevent extensive bowel resection.

In our case, surgery was limited to hematoma evacuation while preserving intestinal continuity. Ileostomy diversion facilitated colonic decompression. Following the procedure, the patient remained under close observation to enable early detection of complications such as hematoma recurrence, full-thickness necrosis, or intestinal perforation. The applied surgical strategy enabled a relatively rapid recovery and restoration of intestinal continuity without defecation-related complications, which would likely have significantly impaired the patient’s quality of life.

**Figure 9.**
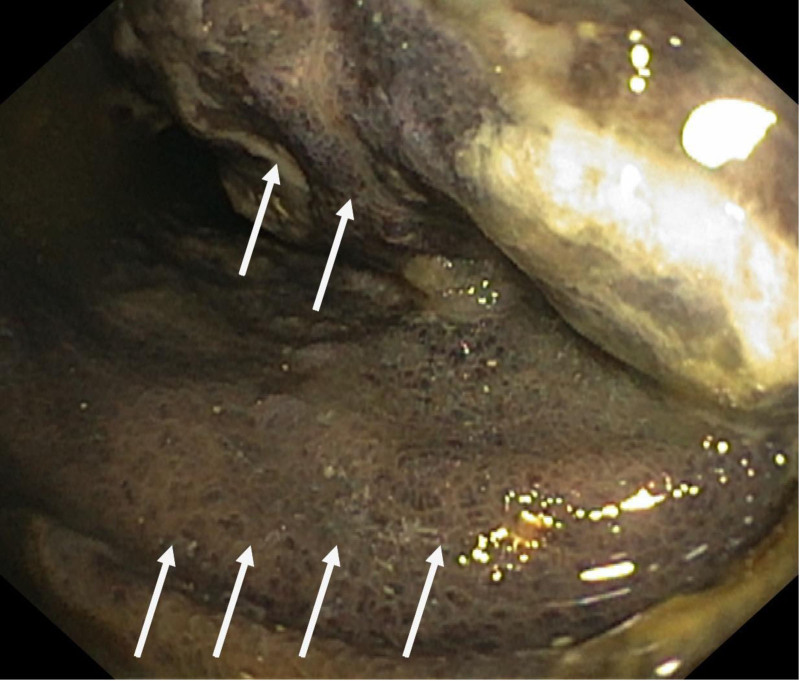
Extensive rectal melanoma (white arrows).

The overall clinical picture, including medical history, physical examination and tests guide us towards the accurate diagnosis. In cases where the symptoms and course are less severe, it is worth considering other pathologies in the differential diagnosis, such as colorectal malignancies and melanoma as a rare and distinct subtype, ischemic colitis, diverticulitis, inflammatory bowel disease, obstructions due to adhesions, volvulus or strictures. Anorectal melanoma, which is a rare form of anal neoplasm, making up only 1.3% of all melanomas.^[16]^ The anorectum is the third most common location of malignant melanoma after the skin and retina.^[17]^ The most common symptoms include obstruction, rectal bleeding, pain, or a sensation of incomplete bowel movement.^[18]^ Anal melanoma most commonly presents as a polypoid mass infiltrating towards the rectal wall and surrounding perirectal tissues. In certain cases, superficial ulceration may be identified.^[19]^ Most tumors are dark in color, which should raise suspicion of malignant melanoma (Fig. 9).

## 4. Conclusion

We have presented a case of an extensive, spontaneous intramural hematoma of the rectum and sigmoid colon in a patient undergoing anticoagulant therapy.

Although these cases are rare, hematomas of the large intestine including rectum, and sigmoid colon should be considered in the differential diagnosis of abdominal pain in anticoagulated patients. Symptoms are nonspecific, so differential diagnosis should also consider colorectal malignancies, ischemic colitis, diverticulitis, inflammatory bowel disease, obstructions due to adhesions, volvulus or strictures.

A timely diagnosis based on clinical assessment, thorough physical examination, and imaging studies can be crucial for further management. In our case, rapid diagnosis allowed for surgical treatment while preserving intestinal continuity.

## Author contributions

**Supervision:** Piotr Wosiewicz.

**Writing – original draft:** Piotr Wosiewicz, Joanna Kula, Paweł Szmigiel, Cezary Rusinowski.

**Writing – review & editing:** Piotr Wosiewicz.
